# Supplementary material for: Relativistic and resonant effects in the ionization of heavy atoms by ultra-intense hard X-rays
Source: Nat Commun. 2018 Oct 10;9:4200. doi: 10.1038/s41467-018-06745-6 (PMC6180123; doi:10.1038/s41467-018-06745-6)
Supplement: Supplementary file 1 — Supplementary Information [file 41467_2018_6745_MOESM1_ESM.pdf]

Supplementary Information

Rudek *et al.*: Relativistic and resonant effects in the ionization of heavy atoms by ultra-intense hard X-rays

SUPPLEMENTARY FIGURES

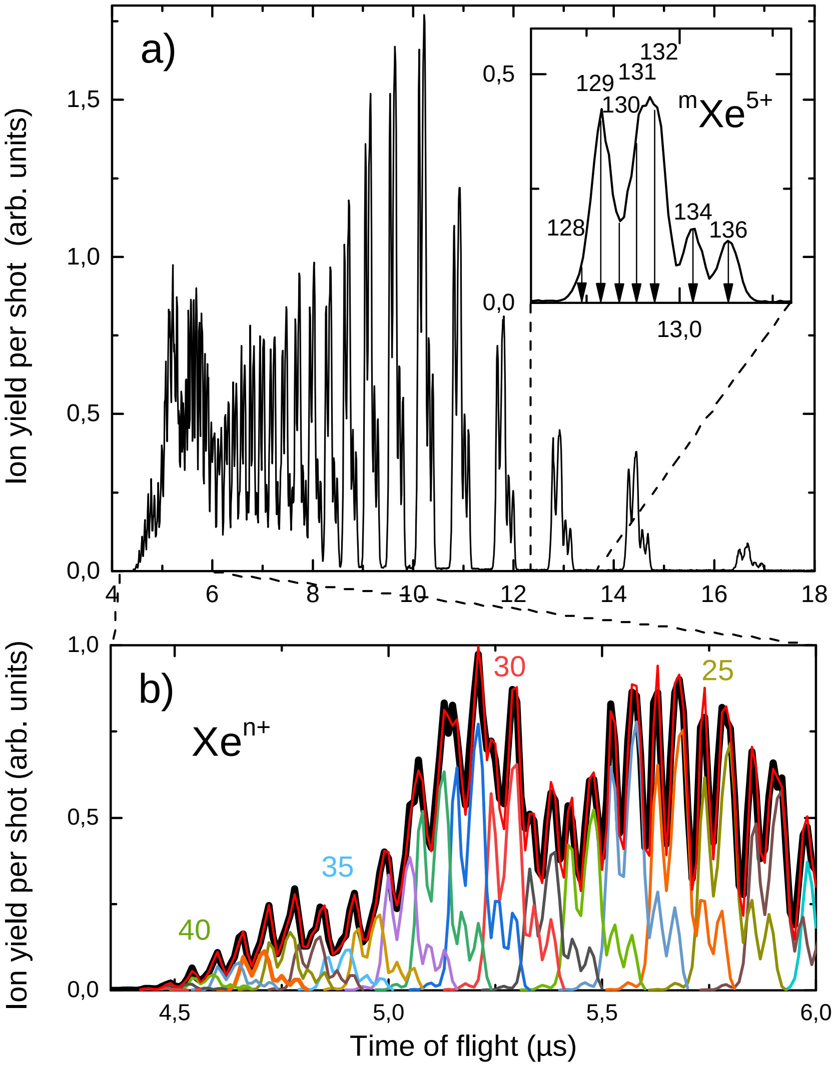

Supplementary Figure 1. **Deconvolution of the time-of-flight spectrum. a)** Ion time-of-flight spectrum of Xe recorded at a photon energy of 5.5 keV with 3.7±0.05 mJ pulse energy. The inset shows Xe^{5+} with the arrows indicating the calculated time of flight (ToF) for seven isotopes. **b)** For decreasing flight times, the peaks of different isotopes of neighboring charge states start to overlap, and a deconvolution based on the natural isotopic abundance is required to determine the ion yield for each charge state, as shown by the colored lines. The ToF of hydrogen and $^{132}Xe^{3+}$ was used to calibrate the ToF spectrum and resulted in the following calibration: ToF$= (\sqrt{m/q} + 0.014)/(3.983 \cdot 10^{-4})$. Every possible ion with charge state q and mass m was fitted with a Gaussian, with the position calculated by the above expression and with the width estimated empirically to linearly depend on the ToF as $\sigma = 0.002 \cdot ToF + 0.1$. The mass m ranged from 128 to 136 to cover all stable Xe isotopes, and the charge q ranged from 3 to 50. The amplitude of each charge state was optimized so that their sum (thin red line) fits the experimental data (thick black line). The method of least squares was applied to optimizes the agreement between fitted and experimental ToF spectrum.

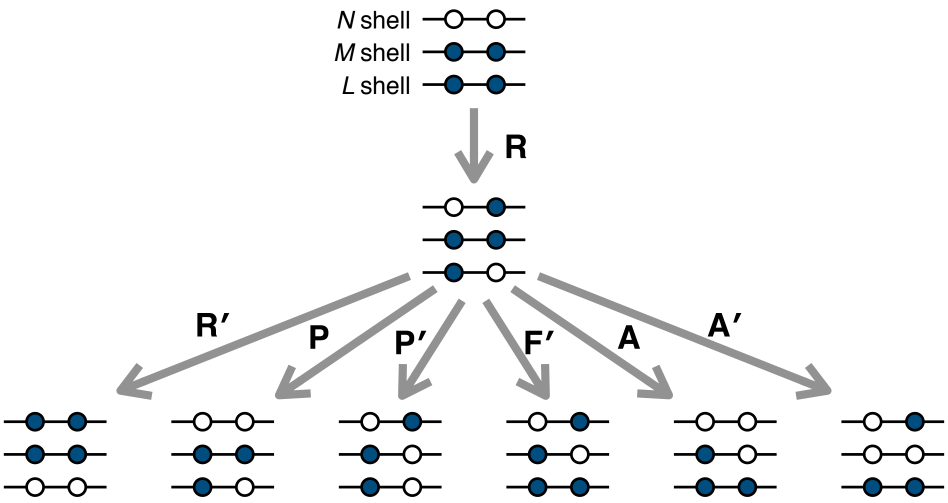

Supplementary Figure 2. Possible processes after resonant excitation. Six different processes may occur after resonantly absorbing one X-ray photon (R: resonant excitation). 1) R′: another resonant excitation, resulting in a multiply excited state; 2) P: photoionization of the excited electron, which corresponds to resonant two-photon absorption; 3) P′: photoionization of an M-shell electron; 4) F′: fluorescence (M-shell $\rightarrow$ L-shell), where the state of the excited electron remains unchanged; 5) A: Auger decay involving the excited electron as a participator; 6) A′: Auger decay leaving the excited electron as a spectator. The latter two pathways, resonant excitation (R) followed by a participator (A) or spectator (A′) Auger decay, are called resonant Auger decay, where the prime indicates that the excited electron remains as a spectator. For high charge states beyond Xe^{26+}, the L-shell (n=2) is the core shell, the M-shell (n=3) is typically the outermost occupied shell, and the N-shell (n=4) is unoccupied. For simplicity, only two electrons and/or holes are depicted in each shell.

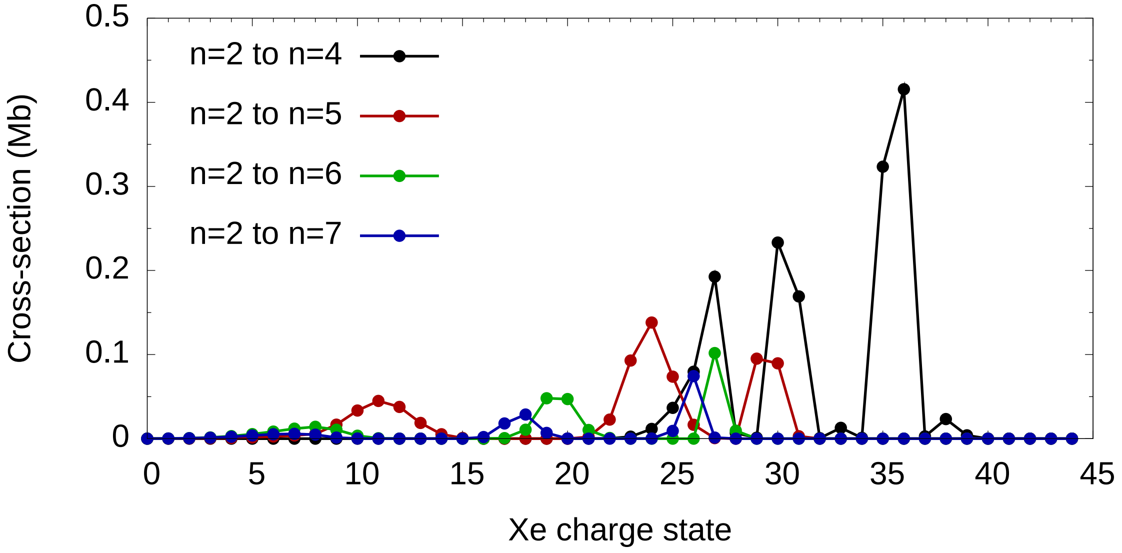

Supplementary Figure 3. Cross-sections for resonant L-shell excitations at 5.5 keV. The cross-sections in Figures 2b and 3 of the main text are total cross-sections obtained by integrating the cross-sections from direct ionization and resonant transitions between all orbitals. This figure breaks down Fig. 2b of the main text into the contributions from electron transition between the $L(n{=}2)$ and the $N(n{=}4)$, $O(n{=}5)$, $P(n{=}6)$ and $Q(n{=}7)$ shells at a photon energy of 5.5 keV. The three largest peaks stem from $2s_{1/2} \rightarrow 4p_{1/2}$ or $4p_{3/2}$, $2p_{1/2} \rightarrow 4d_{3/2}$, and $2p_{3/2} \rightarrow 4d_{5/2}$ transitions. This finding underlines the importance of a relativistic approach, because non-relativistic calculations led to wrong assignments of the participating orbitals in Ref. [1].

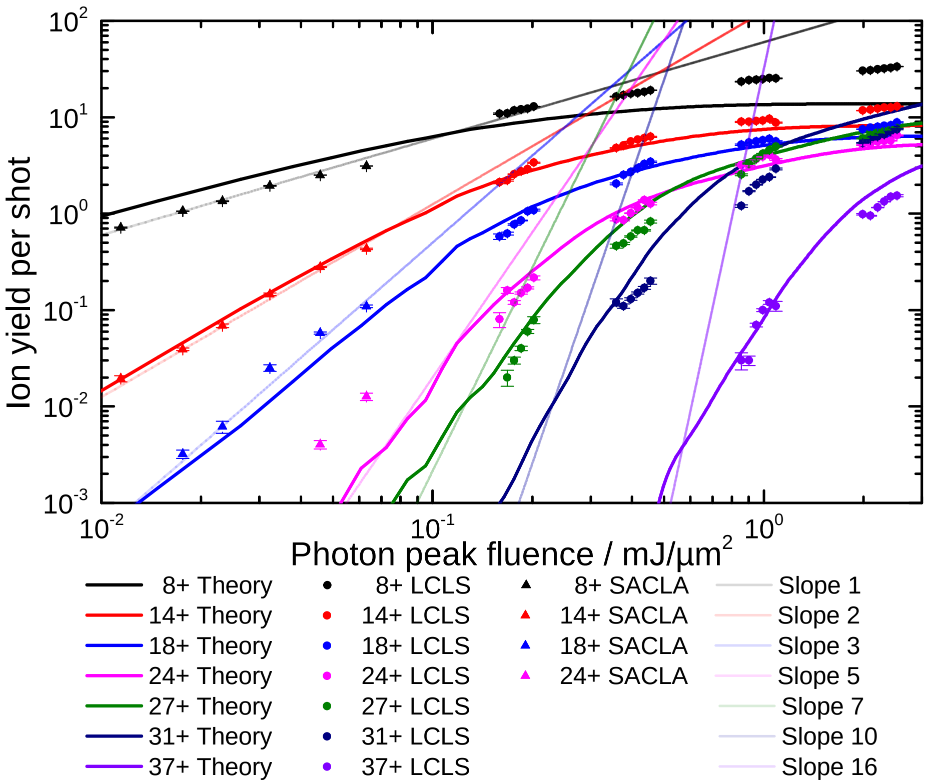

Supplementary Figure 4. **Peak fluence dependence of Xe^{n+} ion yields at 5.5 keV.** In order to cover a wide fluence range, the X-ray beam was attenuated with a set of Si foils (0.02 to 10 mm thickness) by up to two orders of magnitude. In addition, the shot-by-shot pulse energy fluctuations were used to further bin the data into 0.2 mJ intervals. The X-ray pulse energies were measured by gas detectors located upstream of the beamline optics and multiplied by the beamline transmission and the calculated attenuation factor for each Si foil. Closed circles depict the experimental results recorded at LCLS, closed triangles were previously reported for measurements at SACLA [2], and solid lines show theoretical calculations using the method described in this paper. The error bars reflect the uncertainty of the experimental data (1 s.d.) due to the finite counting statistics. Because the SACLA data was taken at different gas pressure, a normalization to the LCLS measurements was required. The theoretical ion yields (solid lines) are scaled by a single factor to match the yield of Xe^{8+} at 50 μJ/μm^2. To guide the eye, straight lines with integer slopes are fitted to the experimental data points before they reach saturation. The values of the slopes are indicated in the figure legend. For further discussion, see Supplementary Discussion.

SUPPLEMENTARY TABLES

Supplementary Table 1. Rates of resonance-induced processes for Xe^{n+}. Calculated rates (in units of fs^{-1}) of the six resonant processes described in Supplementary Figure 2 for Xe^{27+}, Xe^{30+}, and Xe^{36+} at 5.5 keV photon energy, assuming the maximum photon flux used in experiment. The rates quantify the probability of processes after resonant excitation from the ground-state configuration of an ion with the given charge state at a photon energy of 5.5 keV. In all three cases, A$'$ (spectator Auger decay) is the dominant process leading to further ionization. A multiple sequence of RA$'$ will create a multiply excited state, which will then be further ionized by Auger decay or other auto-ionizing transitions. Note that the original REXMI mechanism proposed in Ref. [3] did not include the resonant Auger process as shown here because hardly any electrons were available in the shells between the core and excited levels in the 1.5-keV case.

	Xe^{27+}	Xe^{30+}	Xe^{36+}
R$'$	7.65×10^{-2}	9.38×10^{-3}	1.18×10^{-1}
P	6.43×10^{-3}	2.73×10^{-3}	3.65×10^{-3}
P$'$	8.82×10^{-2}	9.41×10^{-2}	1.07×10^{-1}
F$'$	1.95×10^{-1}	5.45×10^{-1}	2.37×10^{-2}
A	1.84×10^{-2}	3.75×10^{-2}	3.48×10^{-2}
A$'$	4.67×10^{-1}	4.37×10^{-1}	3.50×10^{-1}

SUPPLEMENTARY DISCUSSION

To investigate the fluence dependence of the multiphoton ionization, the experimentally determined ion yields of seven xenon charge states at 5.5 keV photon energy are plotted as a function of peak fluence in Supplementary Figure 4. For a multiphoton process involving the absorption of n photons, the ion yield is proportional to the photoionization rate $\dot{N}_p^{(n)}$, which is defined as $\dot{N}_p^{(n)} = N\sigma^{(n)}(E/\hbar\omega)^n$ according to nth-order perturbation theory, where N denotes the number of target atoms within the interaction volume, $\sigma^{(n)}$ the generalized cross-section for the n-photon process and E the irradiance (fluence divided by pulse length). On a double-logarithmic scale, the slope of the ion yield, drawn as a function of fluence, thus equals the number of absorbed photons. If transitions are saturated, the slope can decrease. Single-photon ionization of neutral Xe at 5.5 keV photon energy, for example, has a cross-section of 0.186 Mb so that for all shots with a photon fluence greater than $5.38 \cdot 10^{10}$ photons/μm^2 (i.e. 47 μJ/μm^2 at 5.5 keV photon energy), one photon is always absorbed. Because Xe^{8+} is generated by such single-photon ionization, its yield curve has a slope of one and saturates at 47 μJ/μm^2. The fact that the theory curves flatten while the experimental yields still increase after that saturation point suggests that the experimental fluence distribution has larger low-fluence areas with increasing peak fluence than modeled. The Xe^{14+} and Xe^{18+} yields are already saturated at the lowest fluence in our experiment. From the SACLA data and the relativistic theory, the number of absorbed photons is estimated to be 2 and slightly lower than 3 for Xe^{14+} and Xe^{18+}, respectively. Xe^{24+} is close to saturation and is fitted to have a slope of 5 in agreement with the calculation in reference [2].

Note that the deformation of attenuating foils by thermal stress at >2 keV was calculated to be negligible and no distortion of the wave front was predicted [4]. However, a degradation of the beam profile was previously observed [5] and might influence the charge state distribution when using the filters.

SUPPLEMENTARY REFERENCES

[1] Ho, P. J., Kanter, E. P. & Young, L. Resonance-mediated atomic ionization dynamics induced by ultraintense x-ray pulses. *Phys. Rev. A* **92**, 063430 (2015).

[2] Fukuzawa, H. *et al.* Deep inner-shell multiphoton ionization by intense x-ray free-electron laser pulses. *Phys. Rev. Lett.* **110**, 173005 (2013).

[3] Rudek, B. *et al.* Ultra-efficient ionization of heavy atoms by intense x-ray free-electron laser pulses. *Nature Photon.* **6**, 858–865 (2012).

[4] Ryutov, D. *et al.* The physics of the gas attenuator for the linac coherent light source (LCLS). Tech. Rep., Lawrence Livermore National Laboratory (LLNL), Livermore, CA (United States) (2009).

[5] Moeller, S. *et al.* Photon beamlines and diagnostics at LCLS. *Nucl. Instrum. Methods Phys. Res., Sect. A* **635**, S6–S11 (2011).
